# Patellofemoral kinematic characteristics in anterior cruciate ligament deficiency and reconstruction

**DOI:** 10.1186/s12891-019-2456-9

**Published:** 2019-02-14

**Authors:** Zhiping Lin, Yangyang Tang, Hongchang Tan, Daozhang Cai

**Affiliations:** 1 The Affiliated Hospital of Guangdong Medical University, Zhanjiang, 524000 China; 2 Guangdong Medical University, Zhanjiang, 524000 China; 3 Department of Orthopedics, Academy of Orthopedics, Guangdong Province, the Third Affiliated Hospital of Southern Medical University, 183 Zhongshan Avenue West, Guangzhou, 510665 China

**Keywords:** Anterior cruciate ligament reconstruction, Patellofemoral joint, Kinematics

## Abstract

**Background:**

It is very important to dynamically evaluate the functional outcome in the knee after anterior cruciate ligament (ACL) reconstruction under physiological weight bearing. The objective of the current study is that we would like to compare the patellofemoral joint kinematics in three ACL status: ACL intact, ACL deficiency, ACL reconstruction.

**Methods:**

Twenty patients with unilateral ACL deficient knees were recruited as preoperative group. Six months after ACL reconstruction, these ten subjects were included as postoperative subjects. Ten normal subjects with healthy knees as the control group. Each subject was asked to walk up a custom set of stairs and a single-plane fluoroscopic imaging system was used to determine the 6DOF kinematics of the injured knees, ACL reconstructed knees, and intact knees.

**Results:**

ACL deficient knees showed reduced patellar flexion angle and reduced distal patellar translation during knee flexion. ACL reconstructed knees showed abnormal patellofemoral joint kinematics compared to ACL intact and ACL deficient knees, exhibiting increased patellar external rotation, lateral tilt, lateral translation during knee flexion.

**Conclusion:**

These findings imply that some alterations persist after ACL deficiency and ACL reconstruction. These abnormal changes will be the onset of degeneration in patellofemoral joint even if the ACL is reconstructed in a way that restores the clinical anteroposterior stability of the knee. Some biomechanical changes should be made to improve the outcome of intervention especially in surgical treatment like ACL reconstruction.

## Background

ACL reconstruction (ACL-R) is one of the main approaches to restore knee stability after ACL injury [1]. In the past few decades, surgeons tend to perform ACL-R on patients with ACL deficiency (ACL-D) rather than conservative treatment [2]. However, in recent years, more conservative treatment is chosen than before, as many studies reported that ACL-R is one of the risk factors inducing osteoarthritis (OA) in patellofemoral joint (PFJ) and tibiofemoral joint (TFJ) [3, 4]. Essentially, the onset of PFJ OA and TFJ OA result from many risk factors after ACL injury, including knee joint biomechanical changes during ACL-R, articular cartilage lesions, meniscus injury and so on [4, 5].

The patellofemoral kinematics are often used to describe the relationship between the patella and the femoral groove. Based on some laboratory studies, patellofemoral kinematics are evaluated through rotational measurement such as internal-external rotation, medial-lateral tilt, and translational measurement such as medial-lateral displacement [6, 7]. Van de Velde et al. have reported that after ACL-R, PFJ kinematics demonstrated some changes such as increased patellar tilt and lateral patellar displacement [8]. The changes of PFJ kinematics will lead to alterations of contact force and contact area in lateral PFJ compartment, thus inducing PFJ cartilage lesions or even the onset of PFJ OA [9]. Van de Velde et al. reported that ACL deficiency resulted in the decrease of knee flexion and the increase of valgus and lateral tilt in patella when subjects performed a single-leg lunge [8]. Resection of the ACL causes an increase of lateral tilt and shift of the patella [5] and decreased patellofemoral contact area and pressure [10]. In ACL-R population, it has been reported that ACL-R restored the patella flexion [8]. The evaluation of PFJ kinematic changes in ACL-D and ACL-R population is mostly reported by vitro cadaveric studies [5, 10]. However, little literature on the evaluation of in vivo dynamic PFJ kinematics in ACL-R population is reported. In fact, it is very important for clinicians to dynamically evaluate the functional outcome in the knee after ACL-R under physiological weight bearing. So, the objective of the current study, was to compare the PFJ kinematics in three ACL status: ACL-I, ACL-D, ACL-R. We will identify the kinematic characteristics in these three different ACL status. We hypothesized that: ACL-D knees show reduced patellar flexion and ACL-R knees show increased lateral tilt and external patellar rotation from extension to 120°of knee flexion.

## Methods

### Subject recruitment

Twenty unilateral ACL deficient patients were recruited as preoperative group (10 men and 10 women,20 to 37 years old, average body mass index, 24.1 ± 4.8 kg/m^2^). Six months after ACL reconstruction, these subjects were included as postoperative subjects. The subjects were included based on intra-operative diagnosis as isolated ACL deficiency. Concomitant knee musculoskeletal disorders or anatomical abnormalities in ACL-D knees were excluded. ACL-D subjects with a history or evidence of injury, or surgery in the contralateral knees were also excluded. For the control group, 10 subjects with healthy knees (6 men and 4 women, 18 to 33 years old, average body mass index, 22.8 ± 6.3 kg/m^2^) were recruited. No knee trauma, pain, and abnormality of movement were found in the healthy subjects. All healthy subjects choose their right knees as the examined knees. Before testing, all the included subjects signed consent form. The institutional review board in authors institute approved the current study design before the initiation of the study.

### Anatomic ACL reconstruction techniques

**Fig. 1:**
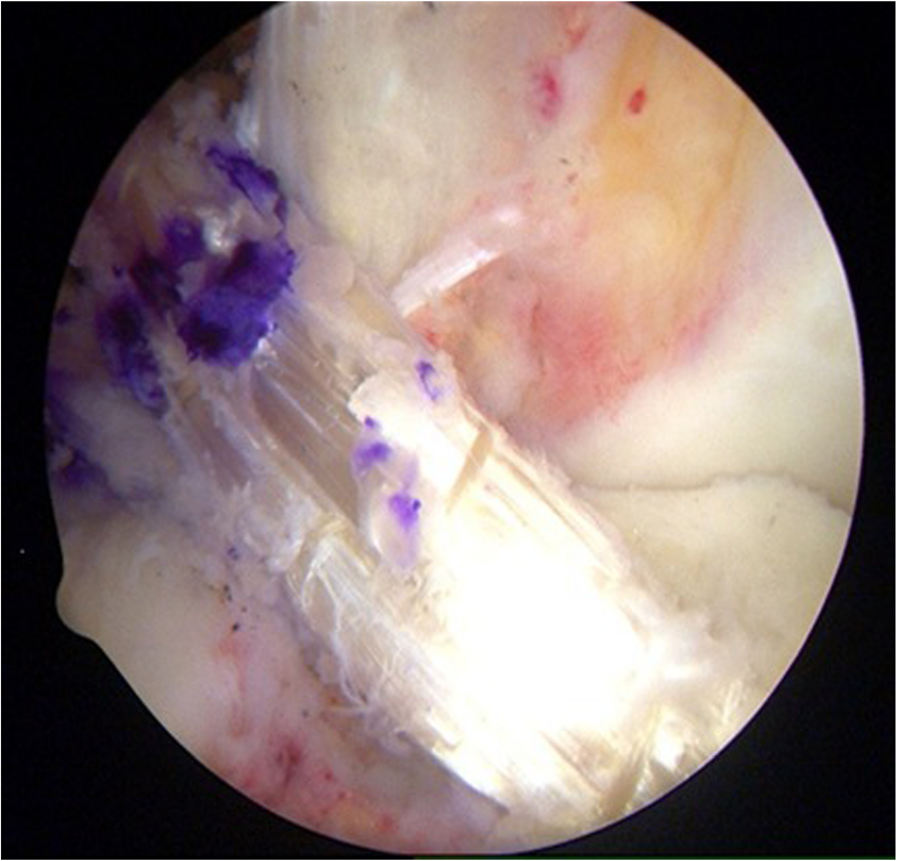
Anatomic Single-Bundle ACL reconstruction

The surgical procedure was standardized and was performed by one experienced surgeon in all patients. The tibial tunnel was addressed first. The centre of the tunnel was placed in line with the anterior horn of the lateral meniscus. All the bone tunnels were drilled 0.5 mm larger that the diameter of the respective grafts, which were between 7.5 and 8.5 mm. The ACL graft consisted of 4-stranded semitendinosus and gracilis tendons. Tibial fixation was achieved with a 7–9 mm interference screw (RCI, Smith & Nephew, Andover, Massachusetts, USA). On the femoral tunnel side, the femoral ACL insertion site was marked with an awl in the shallow aspect of the AM bundle insertion site. The femoral tunnel was predrilled with a 4.0-mm sharp non-cannulated drill. Femoral fixation was achieved with an Endobutton (Acufex, Smith & Nephew, Andover, Massachusetts, USA). Graft tensioning was performed at 10° to 20° of knee flexion (Fig. 1).

### The computed tomography scan and knee modelling reconstruction

Segmentation of computed tomography data (CT, SOMATOM Definition; Siemens, Munich, Germany) was performed for the knee joint. Parallel CT digital images with a thickness of 1 mm without a gap and with a resolution of 512 × 512 pixels were obtained. These data were then imported into solid modeling software Mimics 17.0(Materialise, Leuven, Belgium) to outline the contours of the femur, the patella, and tibia manually. The construction of 3D geometric models of these three bone were done by these outlines.

### Single-plane fluoroscopic imaging of in vivo dynamic knee flexion

**Fig. 2:**
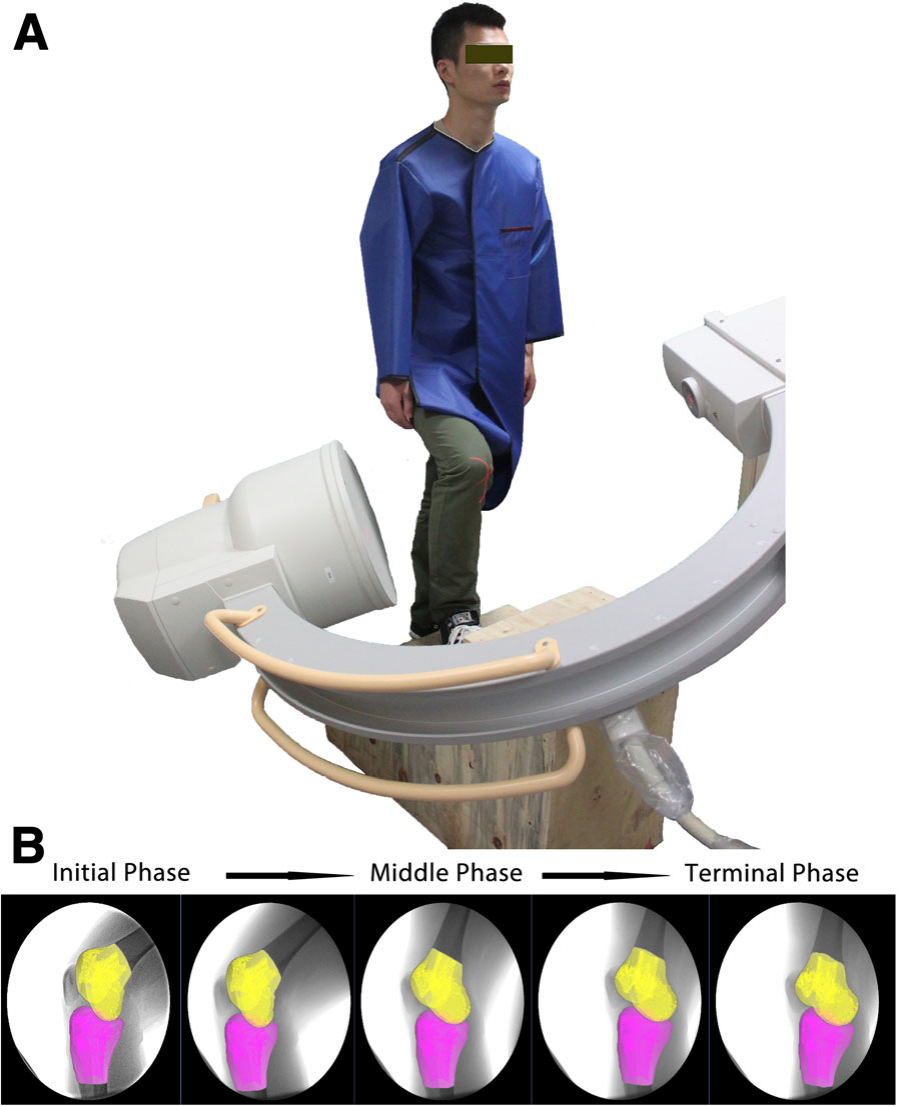
Experimental setup and testing procedure. **a** Measurement of in vivo knee kinematics during ascending stairs by single fluoroscopic imaging system. **b** Virtual reproduction of patellofemoral kinematics during ascending stairs

Next, we used a single-plane fluoroscopic imaging system to investigate the 6 degree of freedom (6 DOF) kinematics in the knee joint. This system has been validated for treadmill gait analysis in our previous published study [11]. Laser-positioning devices, were used to align the target knees within the field of view of the fluoroscopes when subjects were ascending the stairs (Fig. 2a). We asked each subject to walk up a custom set of stairs which was validated in a previous study [11]. This custom set of stairs, with each step 18 mm high, 20 mm deep and 40 mm wide, were designed within published ergonomic recommendations [12, 13]. To reduce postural variations, the subjects’ initial positions were carefully examined by an Orthopaedic surgeon. A rhythmic alarm was used to help the patients ascend the stairs at a fixed pace. No constraint was applied to the knees of subjects while they were ascending the stairs. To simulate normal daily activity, subjects ascended the stairs at a self-selected pace. After measurement, fluoroscopic images were processed in the Digital Imaging and Communications in Medicine format.

Selected fluoroscopic images, identified at a specific posture, were imported into the registration software Virtual_knee1.0 (Medmotion, Guangzhou, China). The actual positions of the image intensifiers of the fluoroscopes were then reproduced (Fig. 2b). Meanwhile, the CT image-based 3D knee models were introduced into the fluoroscopic system. To make the outlines of knee joint segmentation match the osseous outlines captured on the single-plane fluoroscopic images, these models were independently translated and rotated in 6DOF. This process was conducted with an established protocol [10]. The models could be manually rotated using the software (Virtual_knee1.0) with an accuracy of 1.57° for rotation and 0.2 mm for translation [14]. Manual matching was first performed, followed by an automated matching. Previously we measured three times on one subject in a same condition using manual matching technique and measurement was done in 5 subjects by one researcher. The intra-rater reliability of manual matching technique is 0.893. After matching, the knee positions during in vivo weight-bearing activities were reproduced, representing the 6DOF kinematics of the knee for each in vivo posture.

### Patellofemoral kinematics collection

**Fig. 3:**
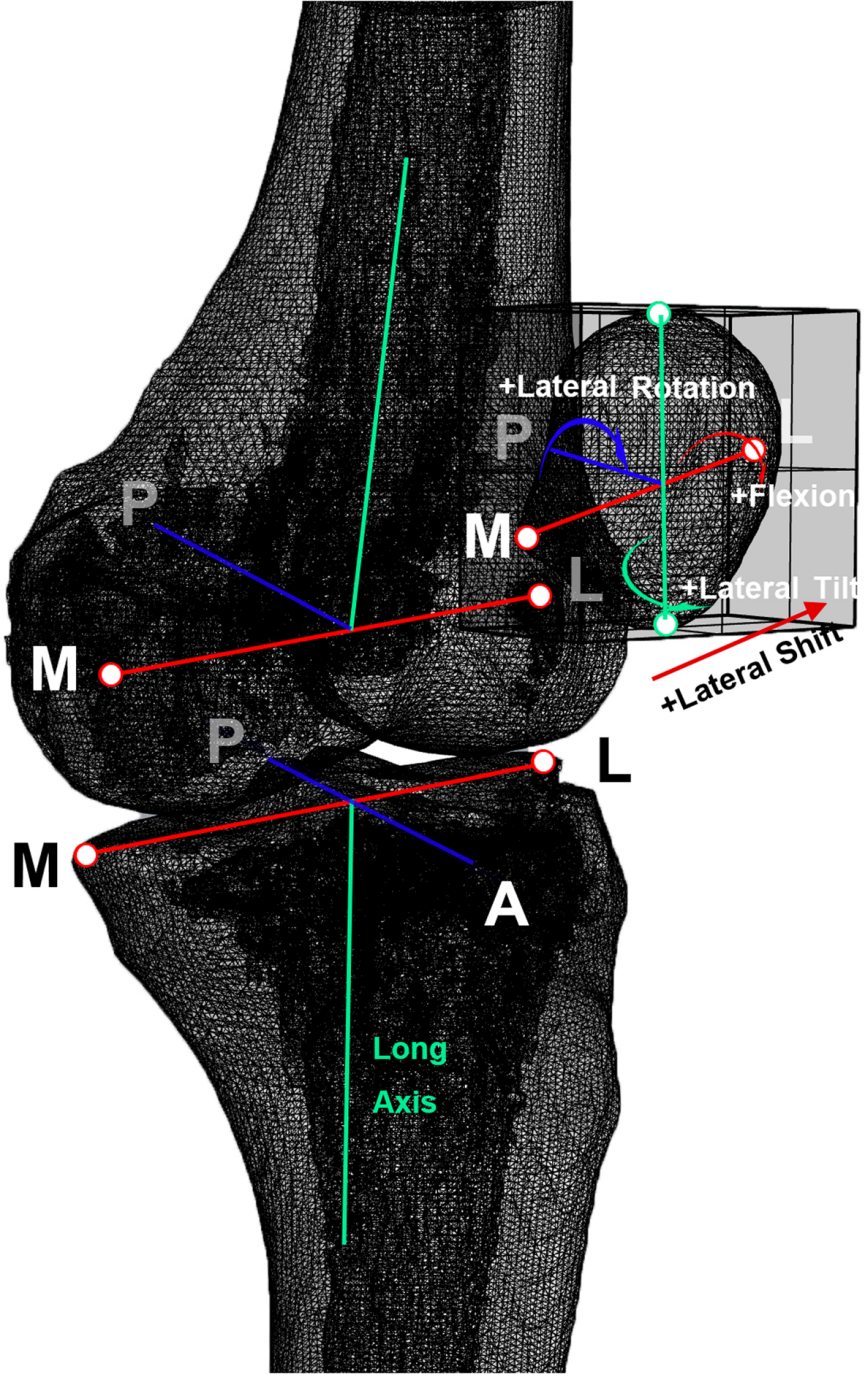
Definition of local femur, patella, and tibia coordinate systems (Footnote) In the femur, the first two points were the prominent points of the medial and lateral femoral epicondyles. The other two points were located paralleling to the wall of the femur shaft. The transepicondylar line was obtained by linking the most pivot points on the medial and lateral condyles. The femoral origin was located at the midpoint of the transepicondylar axis. The line that is parallel to the shaft of the femur was defined as the long axis of the femur. In the patella, the first two points were the most medial and lateral points on the patella; the other two points were the most proximal and distal points on the patella. In the tibia, the first two points were the most pivot points on the medial and lateral tibia plateau. The other two points were located paralleling to the wall of the tibia shaft. The line connecting to the most pivot points on the medial and lateral tibia plateau was defined as the medial-lateral axis, and the midpoint of this line was defined as the origin of the tibial coordinate system. The line that is parallel to the shaft of the femur was defined as the long axis of the femur. Patellofemoral rotation and translation was defined as the motion of the patella centre move with respect to the origin in the femoral coordinate system. Tibiofemoral rotation and translation was defined as the motion of the femoral center move with respect to the origin in the tibia coordinate system

A coordinate system based on a protocol by Suntay et al. [15] was used to calculate the kinematics of knees based on the matched bone models (Fig. 3). The algorithm was developed based on our previous study [16]. A “four-points” method was applied to build coordinate systems in the femur, the patella and the tibia. In the femur, two prominent points located on the medial and lateral femoral epicondyles were selected. Another two points that located parallel to the wall of the femur shaft were selected. The transepicondylar line was obtained by linking the most pivot points on the medial and lateral condyles. The femoral origin was located at the midpoint of the transepicondylar axis. The line that is parallel to the shaft of the femur was defined as the long axis of the femur. In the patella, the first two points were the most medial and lateral points on the patella; the other two points were the most proximal and distal points on the patella. The line connecting to the most medial and lateral points on the patella was defined as the medial-lateral axis, and the midpoint of this line was defined as the origin of the patella. In the tibia, the most external points on sides of the medial and lateral tibia plateau were selected. Another two points that located parallel to the wall of the tibia shaft were selected. The line connecting to the most pivot points on the medial and lateral tibia plateau was defined as the medial-lateral axis, and the midpoint of this line was defined as the origin of the tibial coordinate system. Patellofemoral rotation and translation was defined as the patella center move with respect to the origin in the femur coordinate system We measured the 6 DOF kinematics in the PFJ, including 3 rotational freedom (medial-lateral patellar tilting; medial-lateral patellar rotations; and patellar flexion-extension) and 3 translational freedom (medial-lateral patellar translations; anterior-posterior patellar translations; and proximal-distal patellar translations).

### Data analysis

A two-way repeated ANOVA was used to compare the patellofemoral kinematics. The dependent variables were 6 DOF kinematics in the PFJ as mentioned above. The independent variables were the test conditions (ACL-I, ACL-D and ACL-R) and flexion angles (0°, 30°, 60°, 90°, 120°). We applied the Bonferroni to post hoc multiple comparisons when a statistically significant difference was detected (0.05 significance level). The statistical analysis was performed using commercially available software (SPSS for windows 13.0, Chicago, IL, USA).

## Results

### Patellofemoral kinematics

**Fig. 4:**
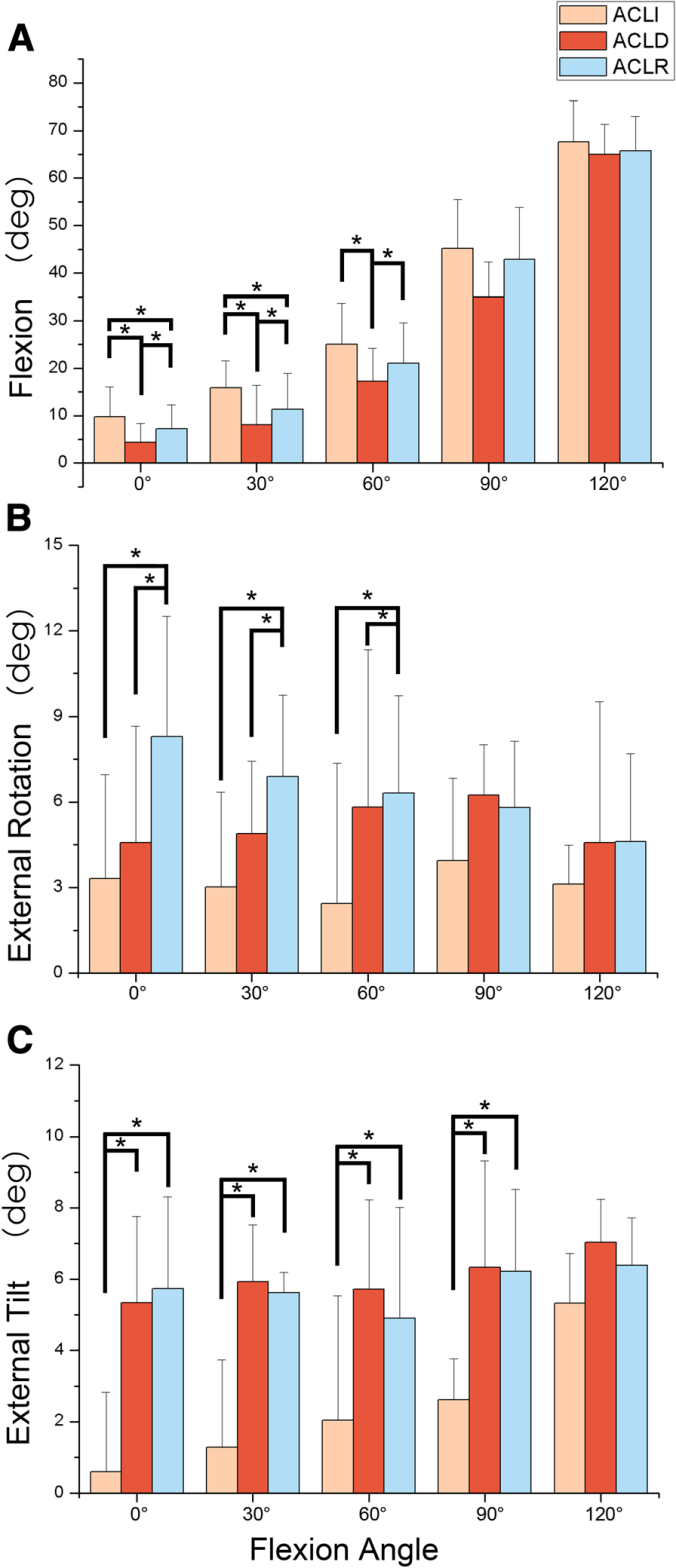
Patellofemoral kinematics (rotations) of ACLI, ACLD, and ACLR knees during ascending stairs. The values represent the motion of the patella with respect to the femur. Asterisk denotes statistically significant difference at *P* < 0.05. **a** Patellar Flexion; **b** Patellar External/Internal rotations; **c** Patellar Internal/External tilt

In ACL-I group, the patellar flexion angle is about a half of knee flexion (Fig. 4a). Patellar flexion angle significantly reduced in ACL-D group compared with other two groups with from 0°~ 60°(*P* < 0.05). In ACL-D group, patellar flexion was 4.3°±. 4.0 at 0° of knee flexion and increased to 64.9 ° ± 6.3 at 120°of knee flexion. Compared to control knees, ACL-R knees showed significant decreased patellar flexion at lower flexion (*P* < 0.05). There is no significant difference detected in these three groups after 60°.

Generally, the patella showed increased external rotation when the knee flexed (Fig. 4b). For internal/external rotation, ACL-R exhibited an increase of about 3~5°in external rotation compared with other two groups at lower flexion (*P* < 0.05). ACL-R group peaked at patellar external flexion (8.3 ± 13.5°) when the knee extended and then continuously decreased. Significant difference was only detected at 60 of knee flexion between ACL-I group and ACL-D group (2.4 ± 7.6° VS 5.8 ± 5.4°).

ACL-D group and ACL-R group showed sharp increased lateral tilt during the whole knee flexion procedure (Fig. 4c). From 0° to 90° ACL-I group revealed a significant increased patellar flexion angle compared with the other two groups (*P* < 0.05) and no significant difference was shown between the ACL-D group and ACL-R group. At 120° of knee flexion, all three groups exhibited highest lateral tilt (5.3 ± 1.4°, 7.0 ± 1.2°, and 6.4 ± 1.3° respectively) in their corresponding ACL status.

**Fig. 5:**
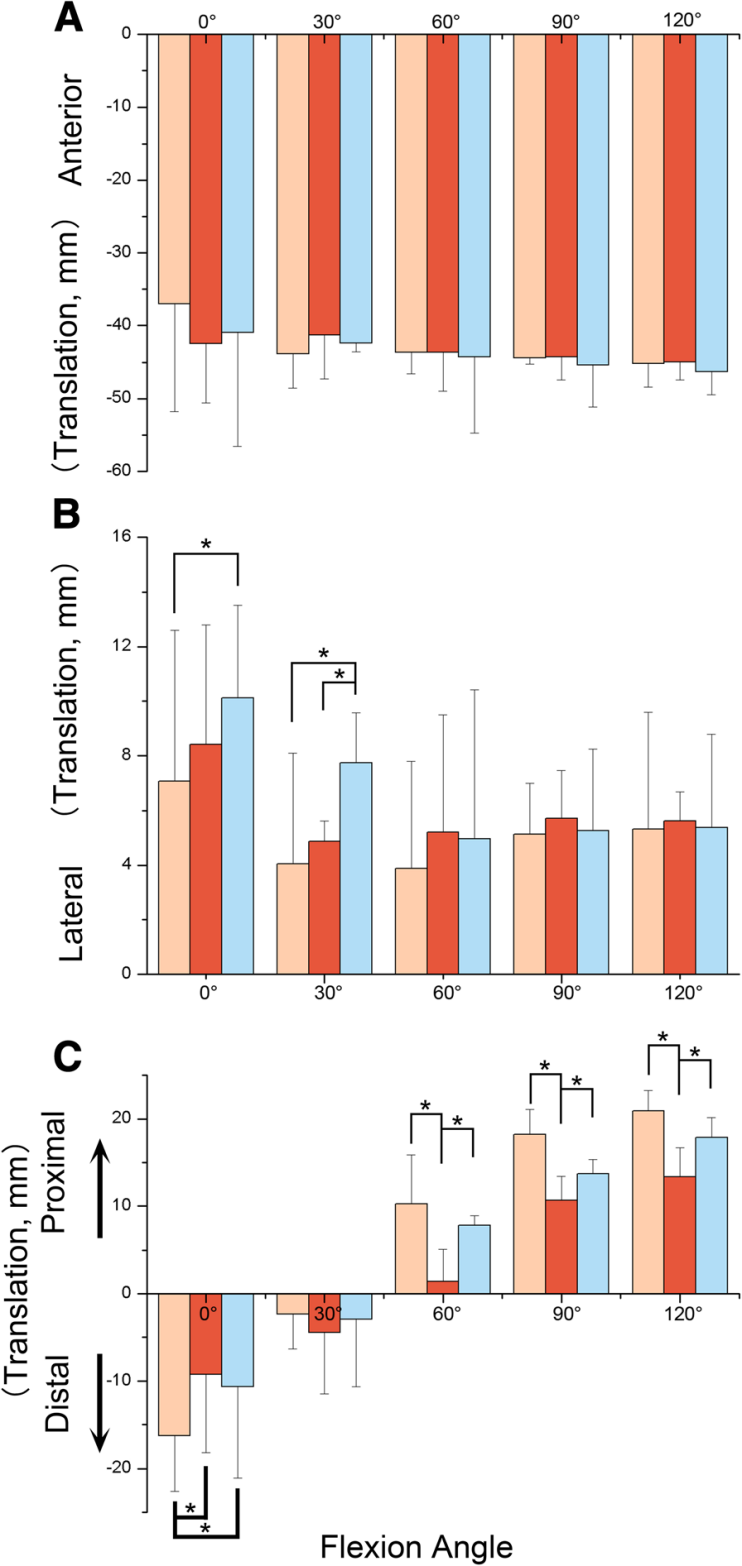
Patellofemoral kinematics (translations) of ACLI, ACLD, and ACLR knees during ascending stairs. The values represent the motion of the patella with respect to the femur. Asterisk denotes statistically significant difference at *P* < 0.05. **a** Patellar Anterior Translations; **b** Patellar Lateral Translations; **c** Patellar Proximal/Distal Translations

In general, the patellar showed anterior patellar translation during the knee flexion (Fig. 5a). No significant difference was found among the groups through the whole measured range, even though ACL-D group and ACL-R group showed more anterior translation compared to control knees.

Compared to control knees, ACL-R group showed more lateral translation (about 3 mm) than the figure from control knees (*P* < 0.05, Fig. 5b). ACL-R group exhibited 10.1 of patellar flexion when the knee extended, followed by decreasing patellar lateral translation as the knee flexed. In particular, ACL-D group showed a significant reduce in lateral translation compared to ACL-R group at 30° of knee flexion (4.8 ± 0.7 mm VS 7.7 ± 1.8 mm).

The patella exhibited a distinguished movement pattern with respect to the description of proximal-distal translation (Fig. 5c). Initially the centre of patella was beneath the femur when the knee extended, up to 16.3 ± 14.7 mm. The patella moved up and was above the femur as the knee extended, up to 20.9 ± 2.3 mm. A significant reduce (about 1.4~7 mm) of distal translation was found in ACL-D group compared with the other groups (*P* < 0.05) when the knee extended. As the knee flexed, similar difference was shown at 60°, 90°, and 120° of knee flexion. Even though no significant proximal-distal translation was detected between ACL-I group and ACL-R group, the latter group showed less proximal patellar translation of about 3 mm.

## Discussion

In the current study, we combined CT reconstruction modelling and single-plane dynamic fluoroscopic imaging to reproduce the 3D position of knees with three ACL status. It enabled us to compare the patellofemoral kinematics in ACL intact, ACL deficient, and ACL reconstructed knees. We collected the kinematic information preoperatively and postoperatively and from controlled group in control subjects’ knees. We performed anatomical single-bundle graft reconstruction with 4-stranded semitendinosus and gracilis tendons. The main findings from our study are that ACL-R showed abnormal PFJ kinematics compared to ACL-I and ACL-D, exhibiting increased patellar external rotation, lateral tilt, lateral translation during knee flexion.

The prevalence of PFJ OA after ACL-R might be underestimated. Approximately 11 to 90% of patients after ACL-R have PFJ OA [17]. Alterations of kinematics and subsequent contact mechanics have been proposed as a possible mechanism in the etiology of PFJ OA after ACL-R. Increased external knee adduction moment was reported 5 years after ACLR to be associated with the development of medial TFJ OA. This altered TFJ biomechanics have an influence on PFJ loading patterns by putting more pressure on the lateral patellar facet and predisposes the PFJ to OA [18]. The abnormal biomechanical adaptation observed in PFJ may impose unfavourable mechanical stress to hamper the natural healing process and may render an inefficient response to ACL rehabilitation. It may account for why approximately 11~ 90% of the patients with ACL deficiency had the possibility of PFJ OA after surgical treatment. However, we need a longitudinal clinical study to investigate the relationship between the altered PFJ kinematics and the presence of PFJ OA including prevalence, regions and area with a long follow-up. In the current study, as the follow-up duration was short and thus PFJ degeneration was not found, we can not justify the relationship between the altered PFJ kinematics and the presence of PFJ OA.

Regarding patellar flexion, the patella showed around half magnitude (0~60°)of that in tibiofemoral flexion during knee flexion. This finding is consistent with results reported in previous studies [8, 19]. Among them, ACL deficiency was shown to significantly disturb the patellar flexion during flexion at lower knee flexion angles. The cause for the decrease may be due to the increased anterior tibial translation. As the patellar tendon is attached on the tibial tubercle, when the tibia moves forward excessively, it results in more patellar parallel to the tibial shaft. This will lead to decrease in patellar flexion. These alterations were not similar to that shown in tibiofemoral flexion in ACL-D knees, as the latter group showed higher flexion angles compared to the intact contralateral side based on the assumption of a higher flexion gait strategy [20]. In the current study, ACL-R knees showed significant decreased patellar flexion at lower flexion (0~60°). These results showed that even though ACL-R can significantly reduce the excessive anterior tibial translation, it could not restore the normal flexion pattern in PFJ. These alterations may be due to altered tibia motion that has a biomechanical connection between the patella and the tibia through the patellar tendon.

With respect to internal-external rotation, generally the patella showed increased external rotation when the knee flexed. In particular, ACL-R exhibited a significant increase of about 3~5° in external rotation compared to other two groups at lower flexion. The patellar tendon is the conjunction between the patella and the tibia. For tibiofemoral kinematics, even though less abnormality of joint kinematics were reported in the ACL-R knees, a normal level of these parameters was not achieved. The kinematic characteristics of the ACL-R knees were more close to those from ACL-deficient knees rather than ACL-intact knees. It indicates that the ACL-R knees showed “under-corrected” pattern after ACL reconstruction [21]. After ACL reconstruction the tibia partially restored the anteroposterior stability and internal-external rotational stability. However, increased external tibial rotation persisted, which leaded to the increase of patellar external rotation.

Abnormal patellar tilt is the most prominent findings in PFJ kinematics in the current study. Both ACL-D group and ACL-R group showed sharp increased lateral tilt during the whole knee flexion procedure. Most studies indicated that after ACL-R, an improved range of motion in tibial external rotation is exhibited, which leads to increased patellar tilt and PFJ load [22–24]. Increased external rotation also results in the increased length of patella tendon and alterations of orientation between the patella tendon and tibia long axis [8]. All of these contributors are likely to lead to the lateral tilt and external rotation of the patella, which predisposes people to PFJ OA initiation or progression.

Changes in patellar translation were not as obvious as those in rotational freedoms. The prominent patellar translation was shown in sagittal plane. In general, the patellar showed anterior patellar translation during the knee flexion. However, no significant difference was found among the groups through the whole measured range. Abnormal translational findings were mainly found in proximal-distal translation. A significant reduce (about 1.4~7 mm) of proximal translation was found in ACL-D group compared to the other groups when the knee extended. As the knee flexed, reduced distal translation was shown at 60°, 90°, and 120° of knee flexion. The reduced distal patellar translation was likely due to the patellar parallel to the tibial shaft. Regarding the medial-lateral translation, significant increased lateral patellar translation was exhibited in ACL-R group, which is understandable as the increased tibial external rotation leads to patellar lateral shift. There was no significant difference found in anteroposterior translation, which was a distinct motion pattern when compared to tibiofemoral kinematics evaluated in ACL-D and ACL-R groups [20].

Compared with some previous studies on PFJ kinematics after ACL-D and ACL-R [8, 19], the novelty of the current study is that the patients and control subjects were asked to perform a stair ascending task which is a very common task in daily activities. Besides, the subjects were asked to ascend stairs at a self-selected pace, which means that the task was physiological, continuous and similar to ascending stairs during daily life. What’s more, the control group in our study was from intact knee population which can be representative of a population with healthy knee kinematics.

### Limitations

There are some limitations in this study. First, a small number of sample size was included. We didn’t perform a statistical power analysis to do sample size calculation as there was no similar study using the same 2D-3D matching technology to evaluate PFJ kinematics. Second, we only performed stair climbing and other functional activities should also be included to evaluate the kinematic alterations of PFJ such as level walking, stair descending, and single leg hop. Third, the mean follow up after ACL-R was about 6 months, which might introduce selective bias in inclusion of subjects. In future study, patients should be followed up at various time intervals to investigate the change in PFJ kinematics over time. Fourth, PFJ kinematics represent the spatial alterations between the patella and the femur. However, as an in-vivo study, some biomechanical parameters such as contact pressure and contact area which are directly associated with biomechanical changes were unable to be collected.

## Conclusion

In conclusion, ACL deficient knees showed reduced patellar flexion angle and reduced distal patellar translation during knee flexion. ACL-R knees showed abnormal PFJ kinematics compared to ACL-I and ACL-D, exhibiting increased patellar external rotation, lateral tilt, lateral translation during knee flexion. These findings imply that some alterations persist after ACL-R. These abnormal changes may result in degeneration in PFJ even if the ACL is reconstructed in a way that restores the clinical anteroposterior stability of the knee. Some biomechanical changes should be made to improve the outcome of intervention especially in surgical treatment such as ACL-R.

## Abbreviations

ACLD: anterior cruciate ligament deficient
ACLI: anterior cruciate ligament intact
ACLR: anterior cruciate ligament reconstruction
OA: Osteoarthritis
PFJ: Patellofemoral joint
TFJ: Tibiofemoral joint
